# RESILIENCE AND GRANDPARENT CAREGIVERS: A LONGITUDINAL ANALYSIS

**DOI:** 10.1093/geroni/igz038.1813

**Published:** 2019-11-08

**Authors:** Bert Hayslip

**Affiliations:** university of north texas, Denton, Texas, United States

## Abstract

Previous work reflecting a shift in views about custodial grandparents has emphasized such persons’ strengths. Such research has specifically indicated that resilience cross sectionally mediates the relationship between the stressfulness of the demands of raising a grandchild and both adjustment-related and parentally relevant outcomes. To explore the dynamics of such relationships in a one-year longitudinal framework, 86 grandparent caregivers completed a variety of measures targeting personal and parental functioning as well as resilience at initial and one-year follow-up occasions. Findings indicated that resilience at T1 predicated (p < .05) the following at T2: parental stress, parental efficacy, health, well-being, depression, grandparent role satisfaction, self-rated life disruption, and grandchild and grandchild behavioral/emotional difficulties. T1 resilience also predicted a T2 index of overall personal resources (better health/social support, less life disruption). At the same time, the following at T1 emerged as antecedents (p < .05) of T2 resilience: parental stress, parental efficacy, grandparent role satisfaction, depression, health, grandchild attachment, and well-being. In addition, overall personal adjustment (higher grandparent role satisfaction, more positive caregiver role appraisal, greater well-being, higher grandchild attachment, less depression) as well as greater overall personal resources each predicted (p < .05) greater T2 resilience. These findings not only extend previous cross-sectional research reinforcing the value of resilience in understanding grandparent caregivers, but also indicate that numerous parental and personal variables may lay the groundwork for the development of resilience, wherein these data also suggest that the relationship between resilience and grandparent caregiver functioning may be bidirectional in nature.

